# Matrix metalloproteinase 28, a novel matrix metalloproteinase, is constitutively expressed in human intervertebral disc tissue and is present in matrix of more degenerated discs

**DOI:** 10.1186/ar2876

**Published:** 2009-12-09

**Authors:** Helen E Gruber, Jane A Ingram, Gretchen L Hoelscher, Natalia Zinchenko, H James Norton, Edward N Hanley

**Affiliations:** 1 Department of Orthopaedic Surgery, Carolinas Medical Center, 1000 Blythe Boulevard, PO Box 32861, Charlotte, NC 28232, USA; 2 Department of Biostatistics, Carolinas Medical Center, 1000 Blythe Boulevard, PO Box 32861, Charlotte, NC 28232, USA

## Abstract

**Introduction:**

The regulation and elevation in expression of the catabolic matrix metalloproteinases (MMPs) is of high importance in the human intervertebral disc since upregulation of these matrix-degrading enzymes results in matrix destruction associated with disc degeneration. MMP28 (epilysin) is a newly discovered MMP believed to play a role in matrix composition and turnover in skin. It is present in basal keratinocytes where its expression is upregulated with wound repair, and in cartilage and synovium where it is upregulated in osteoarthritis. Recent work has shown that mechanical compression can act to modulate expression of MMP28. The expression of MMP28 is unexplored in the intervertebral disc.

**Methods:**

Following approval by our human subjects institutional review board, we employed microarray analyses to evaluate *in vivo *expression of MMP28 and the MMP28 precursor in human disc tissue, and utilized immunohistochemistry to determine cellular and extracellular matrix localization of MMP28 in 35 human disc tissue specimens. The percentage of cells positive for MMP28 immunocytochemical localization was also determined.

**Results:**

The present work documents the expression and presence of MMP28 in cells and extracellular matrix (ECM) of the human intervertebral disc. Gene expression levels in human disc tissue were detectable for both MMP28 and the MMP28 precursor. MMP28 cytoplasmic localization was present in cells of the outer annulus; it was also present in some, but not all, cells of the inner annulus and nucleus. MMP28 was not found in the ECM of healthier Grade I to II discs, but was identified in the ECM of 61% of the more degenerated Grade III to V discs (*P *= 0.0018). There was a significant difference in cellular MMP28 distribution in the disc (*P *= 0.008): the outer annulus showed the largest percentage of cells positive for MMP28 immunolocalization, followed by the inner annulus and then the nucleus. Herniated discs showed a significantly greater proportion of MMP28-positive cells compared with nonherniated discs (*P *= 0.034).

**Conclusions:**

Findings presented here show the first documentation of intervertebral disc expression and production of MMP28. MMP28 was found in both disc cell cytoplasm and in the ECM of more degenerated specimens, with greater cellular localization in the outer annulus and in herniated disc specimens. These findings are important because of the key role of MMPs in disc turnover and homeostasis, and previous indications of a role for this MMP in matrix repair and matrix turnover in other tissues. Our data, which show the presence of MMP28 in human disc tissue, suggest that MMP28 may have a potentially important role in ECM modulation in the healthy and degenerating disc.

## Introduction

The regulation and elevation in expression of the catabolic matrix metalloproteinases (MMPs) is of high importance in the human intervertebral disc since upregulation of these matrix-degrading enzymes results in matrix destruction associated with disc degeneration [1]. Historically, research has focused upon MMP1, MMP2, MMP3, MMP7, MMP8, MMP9, MMP13 and, more recently, MMP19 and MMP10 [2-12].

In the present study, we turned our attention to MMP28 (epilysin), the newest member of the MMP family, discovered in 2001 by Lohi and colleagues [13] and also by Marchenko and Strongin [14]. Related to our intervertebral disc interests, we were especially interested to find data reporting that the induction of MMP28 requires epidermal injury, suggesting a role for MMP28 in extracellular matrix (ECM) homeostasis [15]. Wound healing studies showed that MMP28 was spatially and temporally regulated. Recent work by Renò and colleagues has shown that mechanical compression can act to modulate wound healing and also to modulate expression of MMP28 [16]. Mechanical compression significantly upregulated MMP28 secretion in hypertrophic scars [16].

The closest relative of MMP28 at the amino acid sequence level is MMP19 (which has recently been identified in the human intervertebral disc [11]). MMP28 is a 59 kDa protein, first identified in keratinocytes and testis, and expressed at lower levels in the lung, heart, colon, intestine, bone, kidney, brain and other tissues [13,17]. MMP28 has catalytic activity as an endopeptidase and has the ability to degrade casein [13], and to date this nonspecific substrate for many proteases [18] is the only protein substrate reported for MMP28. The MMP28 protein requires divalent cations for activity, and was shown to be inhibited by a synthetic MMP inhibitor. MMP28 does not include domains characteristic of other MMP subfamilies (the disintegrin and thrombospondin-like regions found in a disintegrin and metalloproteinase and in a disintegrin and metalloproteinase with thrombospondin) or the transmembrane group as found in membrane-type MMPs [13], and the MMP28 promotor has a unique conserved GF-box that is required for basal expression in keratinocytes [19]. Recent work by Werner and colleagues has shown that MMP28 is upregulated during conditions of demyelation, suggesting another *in vivo *role for MMP28 [20].

In the work reported in the present article, we investigated whether MMP28 was expressed in the human intervertebral disc. Our objectives were to determine whether MMP28 and its precursor are expressed *in vivo *in human disc tissue, and to assess the location of MMP28 in disc tissue using immunohistochemistry.

## Materials and methods

### Clinical study population

Experimental study of disc specimens was approved prospectively by the authors' Human Subjects Institutional Review Board. Since disc surgeries are routinely performed in our institution, and surgically removed tissue is discarded, informed consent was not required. The Thompson grading system is used to score disc degeneration over the spectrum of stages from Thompson Grade I (a healthy disc) to discs with advanced degeneration (Thompson Grade V) [21]. Patient specimens were derived from surgical disc procedures performed on individuals with herniated discs and degenerative disc disease. Surgical specimens were transported to the laboratory (less than 30 minutes after surgical removal) in sterile tissue culture medium and were placed in 10% neutral buffered formalin for no longer than 24 hours. Care was taken to remove all granulation tissue and to sample only disc tissue. Donor disc specimens were obtained via the National Cancer Institute Cooperative Human Tissue Network; the specimens were shipped overnight to the laboratory in sterile tissue culture medium and were processed as described below. Specimens were embedded in paraffin without decalcification and were processed for localization of MMP28 as described below.

### *In vivo *gene expression of MMP28 and the MMP28 precursor

Analysis of human disc tissue was carried out as previously described using laser capture microdissection methods [22]. Annulus gene expression analysis used the Affymetrix microarray system. Total RNA was extracted from cells using the TRIzol reagent (Gibco, Carlsbad, CA, USA), reverse transcribed to double-stranded cDNA, subjected to two rounds of transcription, and hybridized to the DNA microarray in the Affymetrix Fluidics Station 400 (Affymetrix, Santa Clara, CA USA). Affymetrix human U133 X3P arrays were used. The GCOS Affymetrix GeneChip Operating System (version 1.2) was used for determining gene expression levels of MMP28 (Affymetrix gene identification number NM_024302.1) and the MMP28 precursor (Affymetrix gene identification number AF219624.1). Gene array data were uploaded to the Gene Expression Omnibus website [GEO:GSE15227] [23].

### Immunolocalization of MMP28

Paraffin sections were cut at 4 μm, collected on PLUS slides (Allegiance, McGaw Park, IL, USA) and dried at 60°C. Sections were deparaffinized in xylene (Allegiance) and rehydrated through graded alcohols (AAPER, Shelbyville, KY, USA) to distilled water. The remainder of the procedure was performed using the Dako Autostainer Plus (Dako, Carpenteria, CA, USA) automated stainer. Endogenous peroxidase was blocked using 3% H_2_O_2 _(Sigma, St Louis, MO, USA). Slides were treated for 5 minutes with Serum-Free Protein Block (Dako), which was not rinsed off the slides prior to application of primary antibody. Slides were incubated for 1 hour with anti-Epilysin (Santa Cruz Biotechnology, Santa Cruz, CA, USA) at a 1:50 dilution. Goat IgG (Vector Laboratories, Burlingame, CA, USA) was used as a negative control. Secondary antibody was biotinylated rabbit anti-goat (Vector Laboratories) for 30 minutes followed by 4+ Streptavidin-HRP Label (Biocare Medical, Concord, CA, USA) for 10 minutes and Dako Autostainer Plus (Dako) for 5 minutes. Slides were removed from the stainer, rinsed in water, counterstained with light green, dehydrated, cleared and mounted with resinous mounting media. Included with each immunolocalization run were a positive control (human testis) and negative controls for each specimen (processed with the absence of the primary antibody). Sections adjacent to those used for MMP28 localization were stained with H & E and Masson trichrome for general histologic features.

The number of cells positive for MMP28 immunolocalization was counted and the percentage of positive cells was determined. For these cell counts, the mean ± standard deviation number of cells counted for the outer annulus specimens was 229 ± 64.0 (n = 5), for the inner annulus specimens was 375 ± 175 (n = 27), and for the nucleus pulposus specimens was 357 ± 160 (n = 3).

### Statistical analyses

Statistical analysis of data utilized standard methods using SAS software (version 11; SAS Institute, Cary, NC, USA), including Fisher's exact test, analysis of variance (ANOVA) and Tukey's test where appropriate, correlation analyses and *t *tests. Gene expression data from microarray studies were tested for normality and were found not to be normally distributed. These data were therefore log-transformed prior to ANOVA analyses.

## Results

### Molecular confirmation of *in vivo *expression of MMP28 and the MMP28 precursor gene in the human disc

**Figure 1:**
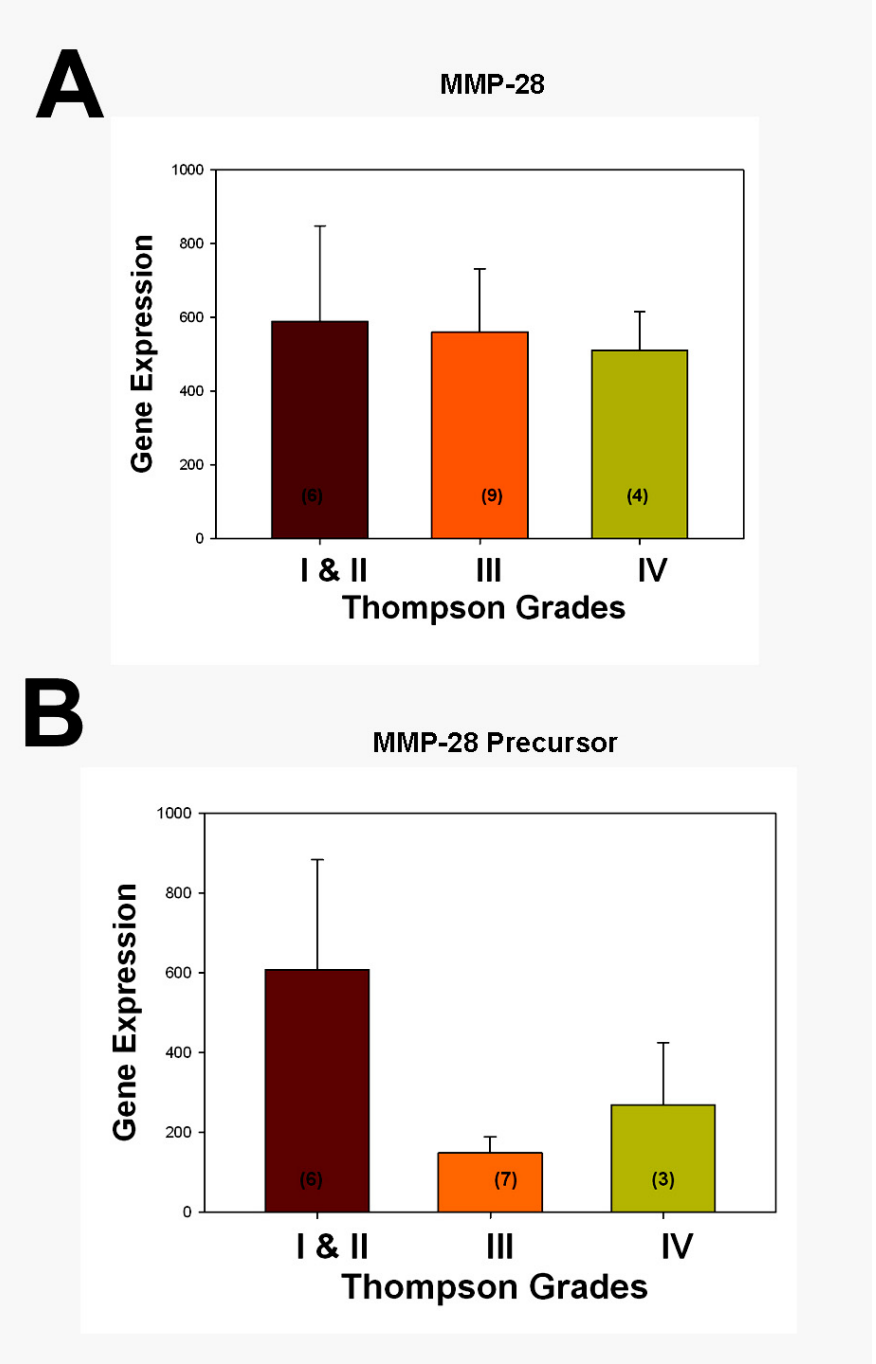
Gene expression for matrix metalloproteinase 28 and precursor *in vivo *in human annulus tissue. Relative gene expression levels for **(a) **matrix metalloproteinase (MMP) 28 and **(b) **MMP28 precursor *in vivo *in human annulus tissue according to grades of disc degeneration. Data presented as the mean ± standard error of the mean. Values inside bars show the number of specimens analyzed for the respective grade of disc tissue.

The *in vivo *expression of MMP28 was analyzed in annulus tissue from 19 surgical disc specimens (six Thompson Grade I to II discs, nine Grade III discs, and four Grade IV discs) as previously described using laser capture microdissection to harvest disc cells from paraffin-embedded tissues and using gene expression analysis with a microarray [22]. The MMP28 precursor gene expression was detected in 16 of the specimens. The results are shown in Figure 1 with data related to the grade of disc degeneration (expressed as mean ± standard error of the mean). ANOVA analysis of log-transformed data showed no significant relationship between gene expression data for MMP28 and the MMP28 precursor and Thompson grades.

### *In vivo *localization of MMP28 in the human intervertebral disc

Table 1 summarizes the demographic features of the patient population studied for immunolocalization of MMP28. Tissue specimens were analyzed surgical specimens or normal donor discs; note that some specimens contained more than one site of disc tissue (outer annulus, inner annulus or nucleus pulposus). Specimens ranged from healthy Thompson Grade I to very degenerated Grade V stages of degeneration.

**Table 1:** Summary of clinical and demographic features in the study population

Subject number	Age (years), gender	Thompson grade	Vertebral level^a^	Herniation	Other information
Young subjects					
1	17, female	I	Lumbar	No	Normal donor
2	10, female	I	Lumbar	No	Normal donor
Adult subjects					
3	21, male	II	L_5 _to S_1_	Yes	Surgical patient
4	50, male	II	L_3 _to L_4_	Yes	Surgical patient
5	46, male	II	L_5 _to S_1_	No	Surgical patient
6	41, male	II	L_5 _to S_1_	Yes	Surgical patient
7	18, female	II	L_4 _to L_5_	Yes	Surgical patient
8	30, male	II	L_3 _to L_4_	No	Normal donor; liver cancer
9	30, male	III	L_4 _to L_5_	Yes	Surgical patient
10	31, male	III	L_5 _to S_1_	Yes	Surgical patient
11	37, male	III	L_5 _to S_1_	Yes	Surgical patient
12	40, female	III	L_4 _to L_5_	No	Surgical patient
13	41, female	III	Lumbar	No	Normal donor
14	38, male	III	L_4 _to L_5_	Yes	Surgical patient
15	40, female	III	L_5 _to S_1_	No	Surgical patient
16	56, female	III	L_5 _to S_1_	Yes	Surgical patient
17	42, male	III	L_5 _to S_1_	No	Surgical patient
18	33, female	III	L_1 _to L_2_	No	Normal donor, pulmonary embolism
19	37, male	3.5	L_4 _to L_5_	Yes	Surgical patient
20	41, male	IV	Lumbar	No	Normal donor, Hodgkin's lymphoma
21	54, male	IV	L_4 _to L_5_	Yes	Surgical patient
22	65, female	IV	L_1 _to L_2_	No	Surgical patient
23	61, female	V	L_4 _to L_5_	No	Surgical patient
24	41, male	V	L_4 _to L_5_	No	Surgical patient
25	72, female	V	L_5 _to S_1_	Yes	Surgical patient
26	39, male	V	L_5 _to S_1_	No	Surgical patient
27	48, male	V	L_3 _to L_4_	No	Surgical patient
28	79, female	V	L_4 _to L_5_	Yes	Surgical patient
29	52, female	V	L_5 _to S_1_	No	Surgical patient

^a^L, lumbar; S, sacral.

**Figure 2:**
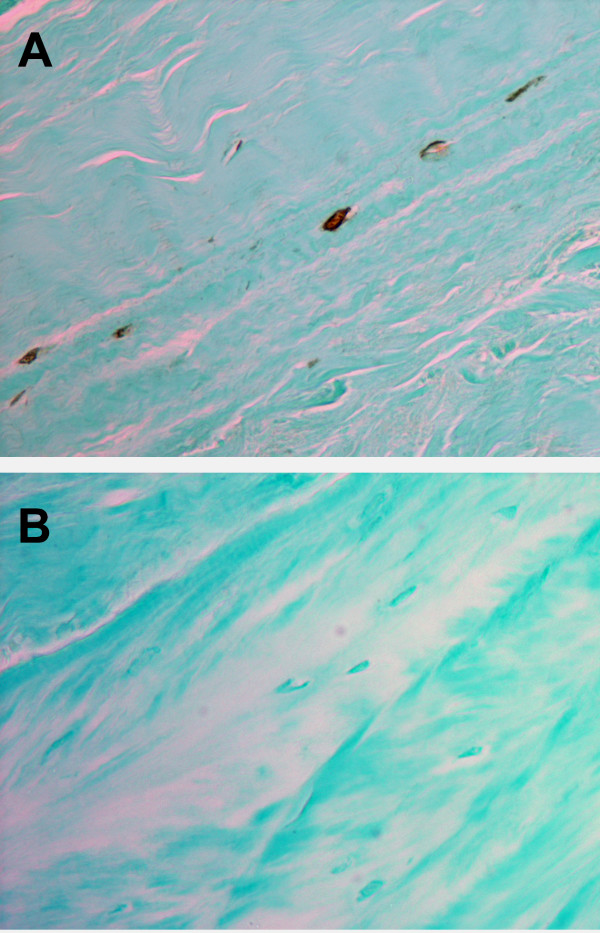
Cytoplasmic localization of matrix metalloproteinase 28 in cells of the outer annulus. **(a)** Cytoplasmic localization of matrix metalloproteinase 28 in spindle-shaped annulus cells in the outer annulus of a Thompson Grade III disc. **(b) **Negative control processed with goat IgG. Magnification: ×450.

**Figure 3:**
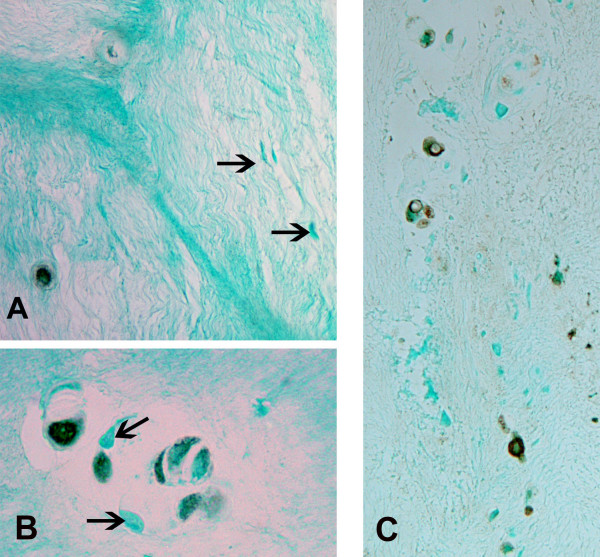
Cytoplasmic localization of matrix metalloproteinase 28 in cells of the inner annulus and nucleus pulposus. **(a) **Cytoplasmic localization of matrix metalloproteinase (MMP) 28 in cells of the inner annulus of a Thompson Grade III disc. Note the negative cells (arrows) that lie near a cell with positive expression. **(b) **Inner annulus cells in clusters in a Thompson Grade III disc. Note the presence of both positive and negative (arrows) cells. **(c) **Some, but not all, cells show MMP28 localization in the nucleus pulposus of this Thompson grade IV disc. Magnification: (a) ×400, (b) ×580, (c) ×360.

MMP28 was present in the vast majority of annulus cells in the outer annulus (characterized by prominent lamellar layers of collagen bundles [24]) (Figure 2). In the inner annulus, localization was present in some, but not all, cells. Negative cells were commonly present near cells with positive localization (Figure 3a). When disc tissue contained clusters of annulus cells, there again was the finding of the presence in some, but not all, cells (Figure 3b). Similarly, some cells in the nucleus pulposus showed the presence of MMP28 (Figure 3c).

MMP28 was not found in the ECM of healthier Grade I to II discs, but was identified in the ECM of 61% of the more degenerated Grade III to V discs (*P *= 0.0018).

**Figure 4:**
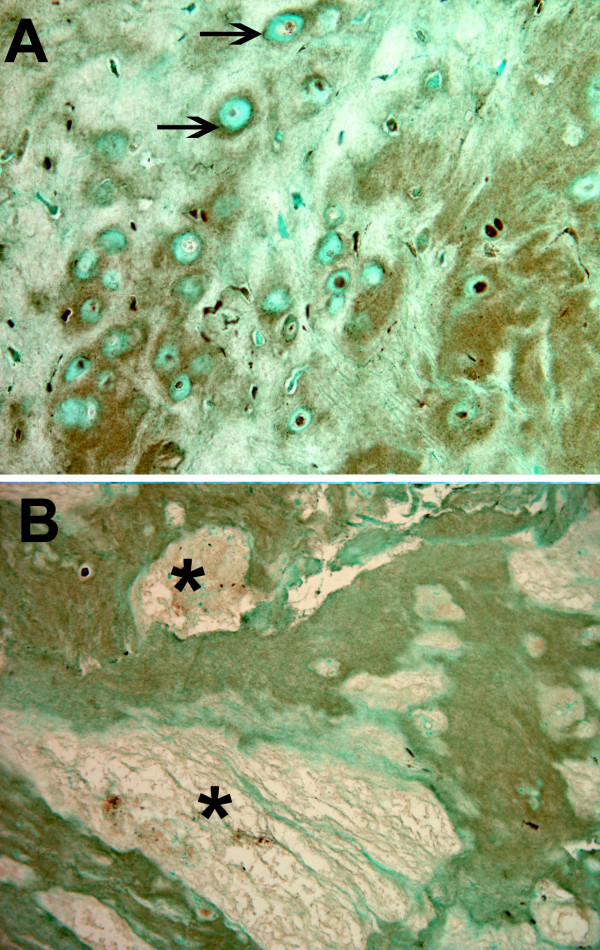
Localization of matrix metalloproteinase 28 in the extracellular matrix of the inner annulus. Localization of matrix metalloproteinase 28 in the extracellular matrix in the inner annulus of a Thompson Grade V disc. Both **(a) **relatively small, localized pericellular (arrows) concentrations and **(b) **diffuse larger localization regions were present in the disc matrix of more degenerated discs. *Nearby presence of focal regions of matrix loss in a Thompson Grade III disc. Magnification: (a) ×325, (b) ×110.

Specimens with higher grades of more advanced degeneration showed focal accumulation of ECM localization of MMP28. Such regions ranged in extent from relative focal, pericellular concentrations around and between cells (Figure 4a) to larger regions of involved matrix that often were near the site of focal matrix loss (Figure 4b, asterisk). Of the specimens evaluated that were Grade III or IV, 43.8% showed positive ECM MMP28 content. All of the specimens that were the most advanced degenerative grade (Grade V) showed MMP28 localization in the ECM.

**Figure 5:**
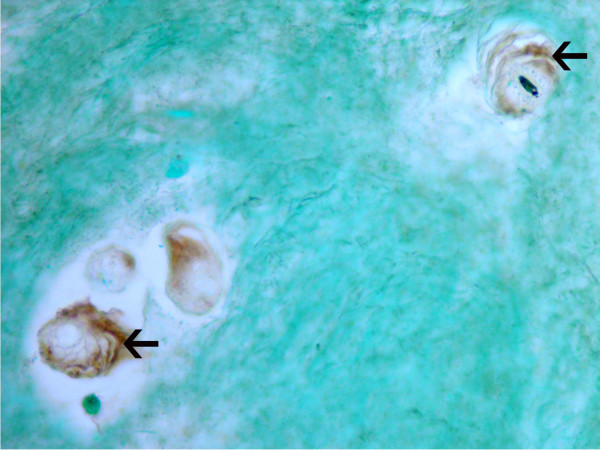
Positive localization of matrix metalloproteinase 28 in the inner annulus. Positive localization of matrix metalloproteinase 28 within the pericellular encapsulation matrix (arrows) of cells in the inner annulus of a Thompson Grade II disc. Magnification: ×465.

Instances were also identified where the encapsulating matrix surrounding disc cells, previously described in histologic studies [25], showed the presence of MMP28 (Figure 5).

**Figure 6:**
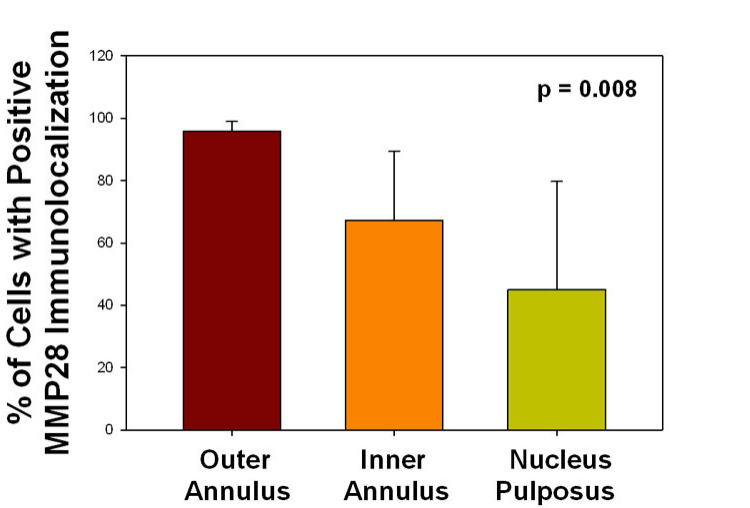
Percentage of cells positive for matrix metalloproteinase 28 immunolocalization. The percentage of cells positive for matrix metalloproteinase (MMP) 28 immunolocalization was quantitatively determined. When analyzed by site, the outer annulus showed the largest percentage of positive cells, followed by the inner annulus and then the nucleus (*P *= 0.008, analysis of variance).

The percentage of cells positive for MMP28 immunolocalization was quantitatively determined in specimens of the outer annulus, the inner annulus or the nucleus pulposus. When analyzed by site, the outer annulus showed the largest percentage of positive cells, followed by the inner annulus and then the nucleus (*P *= 0.008) (Figure 6). Tukey test analysis showed that the percentage of cells positive for MMP28 was significantly greater in the outer annulus compared with the inner annulus and compared with the nucleus (*P *= 0.05).

Analysis was also performed to determine whether the data showed any effect due to the disc site being herniated or nonherniated. Herniated discs showed a significantly greater proportion of MMP28-positive cells (77.0 ± 20.8, n = 20) compared with nonherniated discs (59.3 ± 26.4, n = 15) (*P *= 0.034).

Data were also analyzed in terms of the Thompson grade of the disc. Since our sample sizes were small for some grades, data for healthy Grade I and II discs were pooled, and were analyzed in comparison with Grade III discs and with pooled Grade IV and V discs. ANOVA analysis did not reveal any significant differences in these data (pooled Grade I and II discs, 62.5 ± 29.9%, n = 12; Grade III discs, 70.1 ± 22.4%, n = 11; and Grade IV and V discs, 75.8 ± 2,077%, n = 12). There was also no significant correlation between the percentage of cells positive for MMP28 localization and subject age.

## Discussion

The data presented here show the presence of a new MMP in the human intervertebral disc. A greater percentage of cells positive for MMP28 immunolocalization was present in the outer annulus and in herniated discs. We look forward to future studies designed to experimentally test and define the role of this exciting new MMP in the ECM homeostasis of the healthy disc and its function in the aging and degenerating disc. There are a number of potentially important roles that MMP28 may play in the intervertebral disc; these are discussed below.

### Extracellular matrix localization of MMP28 in the disc

The regulation of MMP activity is now recognized as an important factor in ECM regulation and degradation in the normal and degenerating intervertebral disc. The findings presented here - which show that MMP28 immunolocalization was absent in the ECM of healthier Grade I to II discs, but was present in the ECM of 61% of the more degenerated Grade III to V discs (*P *= 0.0018) - provides novel evidence supporting previous data pointing to a role of MMP28 ECM homeostasis [15]. It is worth commenting here that the deposition of MMP28 within the ECM may represent the result of previous episodes of high MMP28 expression.

Heiskanen and colleagues reported finding overexpressed MMP28 in the insoluble basolateral ECM of cultured confluent epithelial cells [26]. MMP28 was also present in intact basement membrane in wound studies [15], where its expression was seen to be spatially and temporally regulated. Recent research has shown the presence of MMP28 in cartilage and synovium; MMP28 was one of the four most significantly upregulated genes in osteoarthritis [7,27,28], suggesting a role for MMP28 in association with cartilage degradation and/or regeneration. (Unfortunately, the work of Kevorkian and colleagues [28] and that of Davidson and colleagues [27] in osteoarthritis did not include immunohistochemical studies of the cartilage matrix, so we do not know whether ECM localization of MMP28 was present).

### MMP28 and TNFα

Recent studies on matrix synthesis and degradation in the disc have shown that proinflammatory cytokines, such as TNFα, are important cytokines involved in the pathogenesis of disc degeneration. TNFα is elevated in the degenerating disc, induces changes in the biochemical features of discs, notably a loss in proteogycans, stimulates production of nerve growth factor [29], and has been found to regulate MMP3 in nucleus pulposus cells [30]. Saarialho-Kere and colleagues have shown that TNFα induces the expression of MMP28 in cultured primary keratinocytes [15]. MMP28 was not induced, however, by exposure of cells to a number of other cytokines, including basic fibroblast growth factor, epidermal growth factor, granulocyte-macrophage colony-stimulating factor, hepatocyte growth factor, IFNγ, IL-1β (a proinflammatory cytokine also involved in disc degeneration), platelet-derived growth factor, transforming growth factor beta (TGFβ), vascular endothelial growth factor, or insulin-like growth factor 1. Our new finding of the presence of MMP28 in the disc may thus be associated with matrix degradation via involvement of TNFβ.

### MMP28 and transforming growth factor beta

MMP28 may have a connection with another cytokine that has importance in the disc. TGFβ_1_, a cytokine well known for regulating connective tissue metabolism, is present in the disc and is an agent suggested to be associated with changes in the disc ECM during degeneration [31-34]. Illman and colleagues found that lung adenocarcinoma cells that expressed recombinant MMP28 showed this transition with a loss of cell surface E-cadherin [35,36], and showed proteolytic processing of latent TGFβ complexes with a resulting increased level of active TGFβ [35]. Disc tissue with higher concentrations of MMP28 may also have the potential of contributing to elevated TGFβ levels.

### The degenerating disc and herniation and loading

A number of studies have indicated that mechanical load induces MMP expression/activation (see [37] for a review), especially in the aging/degenerating disc [10,38,39]. In our present work, we found a significantly greater proportion of cells with MMP28 immunolocalization in herniated discs compared with nonherniated discs. There is some evidence that mechanical compression can produce a very large release of active MMP28 in heterotrophic scars; these findings may suggest that MMP28 may play a role in restructuring the ECM during loading and healing conditions [16]. It may therefore be important for future studies of MMP28 in the disc to assess its expression in models designed to assess loading and disc matrix failure and herniation. It should also be noted that although the degradative changes in the disc are well recognized in the nucleus pulposus, here we saw a high proportion of cellular immunolocalization of MMP28 in the outer annulus, which may relate to biochemical stress patterns in the outer lamellar regions of degenerating discs.

## Conclusions

The findings presented here show the first documentation of intervertebral disc expression and production of MMP28, a recently identified MMP. Data are important because of the key role of MMPs in disc turnover and homeostasis, and because of previous findings that pointed to a role for this MMP in matrix repair. Both cytoplasmic and ECM localization of MMP28 was present within the disc, with a greater proportion of cells with MMP28 immunolocalization in the outer annulus compared with the inner annulus and nucleus pulposus. A significantly greater cellular localization was also found in herniated discs compared with nonherniated discs. MMP28 was absent in the ECM of healthier Grade I to II discs, but was present in the ECM of 61% of the more degenerated Grade III to V discs (*P *= 0.0018). The results presented here point to the importance of future studies exploring the potential role of MMP28 in disc matrix homeostasis and turnover.

## Abbreviations

ECM: extracellular matrix; H & E: hematoxylin and eosin; IFN: interferon; IL: interleukin; MMP: matrix metalloproteinase; TGFβ: transforming growth factor beta; TNF: tumor necrosis factor.

## Competing interests

The authors declare that they have no competing interests.

## Authors' contributions

HEG and ENH conceived the study and participated in design and coordination. HEG analyzed the immunohistochemistry, performed cell counts, assessed gene expression data, and wrote the manuscript. GLH assisted with gene expression studies. NZ and JAI performed histology and immunocytochemistry. HJN performed all statistical analyses. All authors read and approved the final manuscript.
